# Financial Vulnerability of Dairy Farms Challenged by Johne's Disease to Changes in Farm Payment Support

**DOI:** 10.3389/fvets.2018.00316

**Published:** 2018-12-18

**Authors:** Shailesh Shrestha, Bouda Vosough Ahmadi, Alyson S. Barratt, Steven G. Thomson, Alistair W. Stott

**Affiliations:** ^1^Land Economy, Environment and Society Research Group, Scotland's Rural College, Edinburgh, United Kingdom; ^2^Future Farming Systems Research Group, Scotland's Rural College, Edinburgh, United Kingdom

**Keywords:** Johne's disease, dairy, *paratuberculosis*, farm-level model, economics

## Abstract

Johne's disease is an endemic contagious bacterial infection of ruminants which is prevalent in the United Kingdom and elsewhere. It can lower financial returns on infected farms by reducing farm productivity through output losses and control expenditures. A farm-level analysis of the economics of the disease was conducted taking account of farm variability and different disease prevalence levels. The aim was to assess the financial impacts of a livestock disease on farms and determine their financial vulnerability if farm support payments were to be removed under future policy reforms. A farm-level optimization model, ScotFarm, was used on 50 Scottish dairy farms taken from the Farm Business Survey to determine the impacts of the disease. A counterfactual comparison of five alternative “disease” scenarios with a “no-disease” scenario was carried out to evaluate economic impact of the disease. The extent of a farm's reliance on direct support payments was considered to be an indicator of their financial vulnerability. Under this definition, farms were grouped into three financial vulnerability risk categories; “low risk,” “medium risk,” and “high risk” farms. Results show that farms are estimated to incur a loss of 32% on average of their net profit under a standard disease prevalence level. Farms in the “low risk” and “medium risk” categories were estimated to have a lower financial impact of the disease (22 and 28% reduction on farm net profit, respectively) which, along with their lower reliance on farm direct support payments, indicate they would be more resilient to the disease under future changes in farm payment support. On the contrary, farms in the “high risk” category were estimated to have a reduction of 50% on their farm net profit. A majority of these farms (61%) in the “high risk” category move from being profitable to loss making under the standard disease scenario when farm support payments are removed. Of these, 15% do so because of the impact of the disease. These farms will be more vulnerable if changes were to be made in farm support payments under future agricultural policy reforms.

## Introduction

Estimating the costs of livestock diseases and control measures in terms of losses and benefits is useful for farmers to optimize their managerial decisions, for researchers to understand the impact of diseases on the livestock sector and for policy makers in planning policies to minimize disease impacts on the livestock sector and hence any associated public “bads” (e.g., environment, public health, animal welfare). Numerous studies have examined the financial impacts of livestock diseases and control measures using methods such as regression modeling, economic welfare analysis, cost-benefit analysis, partial budgets, simulation models, dynamic programming, linear programming, partial equilibrium, and general input-output analysis models (1–7). Cost-benefit analyses, general input-output analysis and partial budget models work at the farm-level but are often focused only on disease/no-disease conditions of a farm to compare and determine the net effect of diseases on the financial performance of healthy vs. infected herds. These techniques typically isolate a single activity on a farm and assume no interlinking effects between farm activities. In other words, such techniques rarely consider the farming system as a whole when analyzing the economic impact of diseases or of control measures. Many of these studies which used welfare analysis, regression and simulation models include interlinked activities within a farm to determine average losses on the farm and generate a national estimate based on those figures. These studies, although based on management and production variability within a farm, did not include the variabilities between farms which limits the insight gained from a national-level analysis for individual farmers and also for policy makers dealing with agricultural support policies in planning their farm and sector strategies.

Another issue with these commonly used methods is that they often only consider an average prevalence level for all farm types to generalize the economic impact of a disease. Due to the farm variability that exists between different farm types, impacts of a disease and farm responses to that disease are likely to vary from farm to farm. The economic vulnerability of a farm to a disease not only depends on farm resources, farm management practices, and production efficiency but also to biosecurity measures and the extent of exposure to that disease. Many economic studies examining the impact of external shocks (like policy reforms and climate change) support the importance of farm variability (8–12). These studies highlight that some farms are more capable of responding to shocks than other farms. We test the hypothesis that the same argument is true on farms under a disease scenario. Some farms may survive greater exposure to a disease whereas others may require different strategies to minimize their net effects.

There are many impact assessment studies where farm variabilities are highlighted using farm-level models (5, 8, 9, 11, 13–18). The advantage of this method is that it takes into account all the biophysical and financial characteristics and interactions between different activities of the whole farming system to assess the losses incurred due to external shocks. This also allows analysis of the financial vulnerability and resilience of a farm. A number of studies examined vulnerability of agricultural farms under price changes and agricultural policy reforms (9, 19–21). They show that any adverse changes in market prices and support payments can significantly expose it to economic vulnerability. This paper, hence, considers a holistic farm-level modeling technique which is a useful tool to incorporate farm variability into the economic assessment of livestock diseases. We use a farm-level economic model, ScotFarm, under five alternative disease prevalence scenarios of Johne's disease and examine their financial impacts on Scottish dairy farms at the farm-level.

Johne's disease is an endemic contagious bacterial infection of ruminants caused by *Mycobacterium avium* subspecies *paratuberculosis* and is prevalent in the UK, including Scotland (22). It can reduce farm profit by imposing production losses on infected animals. The production impacts of Johne's disease can result in reduced milk yield without offsetting proportional reductions in feed consumption, increased culling, increased calving interval and infertility as well as increased disease control costs resulting in an economic loss in affected herds. The disease manifests itself within an animal in two stages: a subclinical stage and a clinical stage. At the clinical stage, infected animals show visible disease symptoms and hence it is easier for farmers to diagnose the disease and take necessary actions to reduce production losses. The subclinical stage is a long latent-period where an infected animal does not show observable symptoms. Accurate testing and diagnosing of subclinical cases is very difficult (23) because there is not a reliable test currently available to identify the disease at this stage. This means that a subclinical form of the disease can stay on farm without detection for a long time, thus remaining as a main contributor to production and economic losses and also as a continuous source of the disease spread. Culling and replacing detected cases of infected animals are the main strategies for controlling the disease on-farm.

In this paper, we aim to explore the financial impact of Johne's disease on Scottish dairy farms based on variability on their reliance on farm support payments. We assume that all other financial parameters such as market prices, off farm and diversification incomes remain constant. We consider financial security provided by farm direct support payment as a measure to determine a farm's financial vulnerability given their Johne's disease status. Hence, farms were grouped in different categories of economic vulnerability based on the scale of their reliance on support payments to stay profitable. We then examine the differences in impact of Johne's disease on farms in these different vulnerability groups. We focus on these impacts because of the possibility of change in farm support payments in the UK after Brexit. Similar pressures are on support mechanisms in other western agricultural systems in the world whether that be due to reforms in the Farm Bill in the US or the Common Agricultural Policy in the European Union. All such changes are likely to alter the impacts and importance of endemic disease outbreaks and their control.

## Materials and Methods

### ScotFarm Model

ScotFarm^1^ is a farm-level linear programing model that optimizes financial margins of a farm within its bio-physical constraints. The model maximizes farm net profit which is the sum of gross margins from all farm activities and farm support payments such as Basic Payments Scheme (24) and Less Favorable Area Scheme (25), minus fixed costs (FC). The general mathematical formulation of maximizing farm net profit for the dairy module is as follows:

$$\begin{array}{l} \underset{{\text{x}}_{f,i}\geq 0}{\text{Max}}Z_{f}=\sum \left[gm_{f,i}\right]x_{f,i}+S_{f}+LFAS_{f}-FC_{f} \end{array}$$

Subject to

$$\begin{array}{l} \underset{i}{\sum }A_{f,i}x_{f,i}\leq b_{f}\; \end{array}$$

Where *Z* denotes optimized net profit of all activities from all the enterprises of a farm; *gm* represents gross margin of activities; index *i* denotes agricultural activities including livestock and crop while *f* denotes individual farms; *x*_*f, i*_ is the non-negative activity level in hectares or heads of farm *f* activity *I*; *S* represents farm direct support payment^2^; *LFAS* is the Less Favorable Area Scheme payment; *FC* is total fixed costs; *A* is an input–output coefficient for activity *x*; and *b* denotes limited farm resources.

Dairy gross margin (*gm*_*f, i*_) is estimated as follows:

$$\begin{array}{l} gm_{f,i}=p_{f,i}y_{f,i}+mcp_{f,i}mc_{f,i}+dp_{f,i}cd_{f,i}-hp_{f,i}h_{f,i}\\ \;-\sum VC_{f,i}-\sum NC_{f,i} \end{array}$$

Where *p* denotes milk price, *y* represents total milk yield; *mcp* denotes the price of male calves; *mc* denotes the number of male calves sold; *dp* denotes the price of culled dairy cows; *cd* denotes the number of culled dairy cows; *hp* represents price of purchased heifers; *h* denotes the number of purchased heifers; *VC* represents variable costs [including labor, veterinary, and AI (Artificial Insemination) costs] of dairy cows excluding feedstuffs and *NC* represents feed costs which includes purchased concentrate and home-grown or purchased grass silage.

Total milk production is the summation of milk produced by all lactating cows and assumed to be sold in the market. There is no consideration for spillage, discards, or own consumption. The model assumes a 4-year lactation cycle where 25% of lactating dairy animals are culled each year and replaced by either own produced or bought-in heifers. The resources such as labor and feed required is then determined based on number of animals on farm each year. The model spans 15 years, capturing herd dynamics, dairy cycle and farm management changes. The results are then averaged over the middle 9 years to minimize starting and terminal biasness of the linear programming technique. We assume constant disease prevalence across years within a given scenario. The model and methodology have been used in a number of earlier studies (11, 26, 27).

### Data

Farm level data of 50 Scottish dairy farms were obtained from the Farm Business Survey, FBS (28). The survey collects physical as well as financial information from sampled farms. The data used in the model included physical data, such as: agricultural utilized area under temporary and permanent grass, grass silage, crops and rough grazing; number of cows; crops produced; labor availability; milk yield per cow; and financial data such as variable costs (such as labor, AI, veterinary costs), fixed costs (such as machinery, building, fuel, taxes etc.), prices (such as milk, heifer, and feed prices) and farm support payments (Basic Payment Scheme and LFAS).

### Disease Impact

Johne's disease has several detrimental effects on productivity of dairy cows and a dairy herd in general. Milk yield is suppressed as a result of adverse effects on the digestive tract in both clinical and subclinical cases. Extra culling is required to replace clinically infected animals, which increases replacement costs on the farm. A higher culling rate also imposes additional indirect costs such as reduced opportunity for selection of superior genotypes and increased use of resources for animal growth at the expense of milk production. Presence of the disease on-farm also requires extra veterinary costs per animal and associated opportunity costs of farm labor. The estimates on production loss, culling rate, and additional costs are taken from a Markov-chain epidemiological model (4, 7).

Table 1 presents assumptions used under the five alternative prevalence scenarios (i.e., PL1, PL2, PL3, PL4, and PL5). Prevalence scenario PL2 is based on an estimated prevalence (29) and is considered to be the “*standard disease condition*” scenario. Other alternative prevalence scenarios are estimated to examine a range of values suggested in other studies (7, 30, 31). Parameters affected are increased culling rate at a farm level, and milk yield loss and additional costs at an individual animal level. Additional costs include the sum of opportunity costs associated with the loss of a cow on a superior lactation curve, reduced longevity, additional replacement costs and extra veterinary costs (4). These costs are assumed specifically to be related to sub-clinical cases and hence are used at individual animal level in the model.

**Table 1:** Farm-level assumptions used under the five alternative prevalence scenarios for the number of clinical and sub-clinical dairy cow cases, the farm's total culling rate, milk yield loss and additional costs.

**Prevalence scenarios**	**Percentage of infected animals**		**Culling rate (%)**	**Milk yield loss (%)**	**Additional Johne's disease related costs (£/cow/yr)**
	**Clinical**	**Subclinical**			
PL1	1.0	6.5	26.04	0.88	31.91
PL2^*^	2.0	15.5	27.09	2.02	82.08
PL3	3.0	17.0	28.10	2.38	98.24
PL4	4.0	18.5	29.11	2.74	114.33
PL5	5.0	22.5	30.14	3.34	132.15

* *PL2 is considered the existing level of prevalence in the UK (29)*.

### Model Runs and Prevalence Scenarios

The ScotFarm model was run on a “no-disease” scenario and five alternative “disease” scenarios. The “no-disease” scenario uses farm information from the FBS survey data and assumes all of the sampled farms to be Johne's disease-free. Under the “disease” scenarios, all clinically infected animals are culled (i.e., a culling rate of 100%) but only 18% (4) of subclinical cases are culled. These clinical and subclinical culling rates are in addition to the 25% voluntary culling and replacement rates assumed in the model. The percentage reductions in milk yield and additional costs are generated by the Markov chain model. A counterfactual comparison of the “disease” scenarios against the “no-disease” scenario at a farm level is then made to evaluate economic impact of the disease under alternative prevalence levels. A simple analysis of variance (ANOVA) is conducted to test the difference between farms in three risk categories.

Farms are categorized into three groups of financial vulnerability based on the ratio of farm direct support payment (*S*) received to their net profit excluding farm payments in the “no-disease” scenario. These categories are: **(**i) “low risk”—where the ratio is < l5%; **(**ii) “medium risk”—where the ratio is >5% but < 25% and “high risk”—where the ratio is >25%. Further analysis is carried out based on these three financial vulnerability categories.

## Results

**Figure 1:**
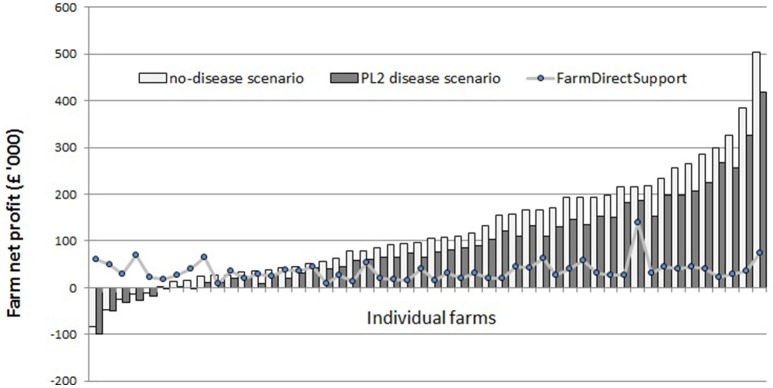
Annual farm net profits under the “no-disease” and the PL2 scenarios for individual farms (*n* = 50) ranked from least to most profitable.

**Figure 2:**
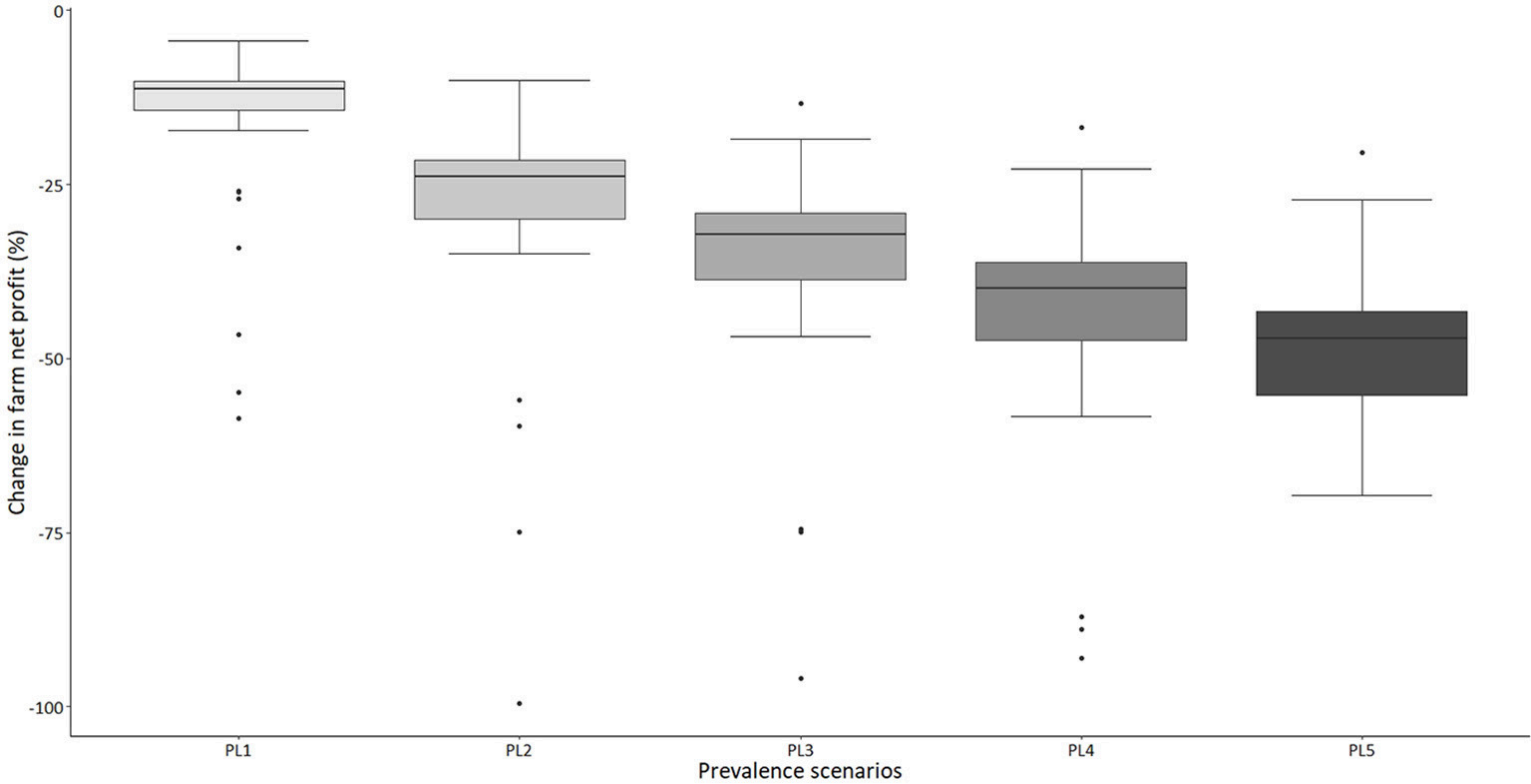
A box-plot representing percentage change in annual farm net profit under five alternative disease scenarios compared to the “no-disease” scenario (outliers are denoted by solid dots, median is the thick black line, the 25 and 75th percentile represented by top and bottom edge of box and the maximum and minimum value are represented by top and bottom whisker, respectively).

The model results suggest that around 90% of the sampled dairy farms make positive net profit per year under “no-disease” conditions (Figure 1). However, there is a large variability in net profit between individual farms. Under the “standard disease condition” PL2 scenario, farm net profit is reduced by 32% on average. The range of impact on farm net profit over all farms varies from −6 to −64% (Figure 2). Reduction in farm net profit increases as the disease prevalence level increases from PL2 to PL5, with the largest reduction in net profit (59% on average) observed under the PL5 scenario. Figure 1 also shows the amount of direct payment received by farms. The majority of farms (80%) receive around £36,168 in direct payments on average per year.

**Figure 3:**
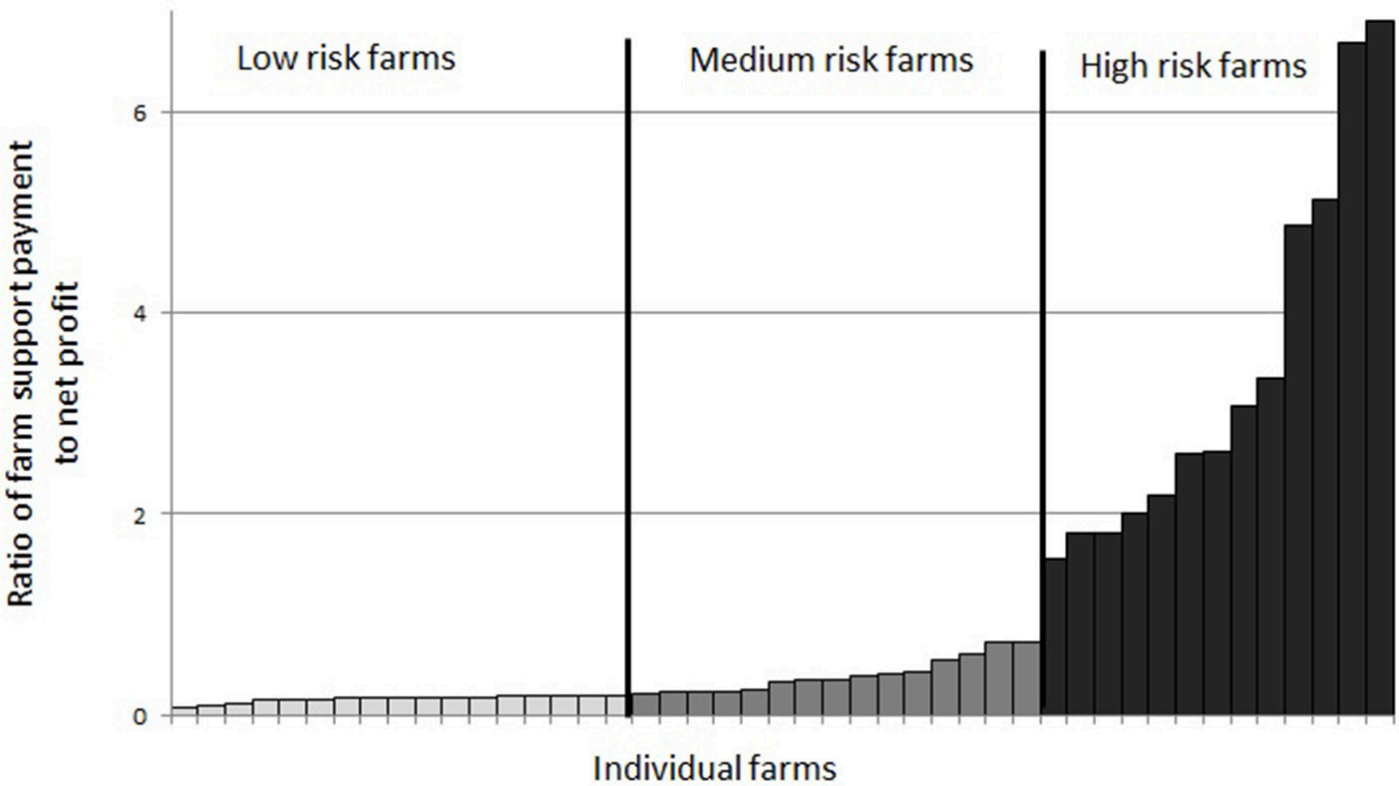
Grouping of farms in financial vulnerability risk categories based on the ratio of farm direct payment (*S*) to farm net profit (*Z*) under the “no-disease” scenario.

Farms (90%) which were making profit in the “no-disease” scenario were categorized into “low risk,” “medium risk,” and “high risk” categories as shown in Figure 3. There were 38% of farms in the “low risk” category, 33% farms in the “medium risk” category and 29% farms in the “high risk” category.

The reduction in farm net profit within these three financial vulnerability risk categories under the five alternative “disease” scenarios compared to the “no-disease” scenario are presented in Table 2. In absolute terms, the reductions in net profit are higher in the “low-risk” category than in the other two financial risk categories under all disease scenarios. For example, under disease scenario PL2, an average “low-risk” farm will lose £43,741 whereas on average “high-risk” farm will only lose around £15,204 in net profit. However, the percentage change in net profit for “high risk” farms is substantially higher than corresponding average percentage change for the other two risk categories, under all disease scenarios. For instance, under PL2, the percentage change for an average farm in the “high risk” category is almost 2-times that of an average farm in “medium risk” or “low risk” categories.

**Table 2:** Average change in annual farm net profit under “disease scenarios” PL1 through PL5 compared to the “no-disease” scenario (Johne's disease free farm; not shown) for farms in different risk categories.

**Prevalence scenarios**	**Absolute change per farm (£) (per cow in parenthesis)**			**Percentage change (%)**		
	**Low risk**	**Medium risk**	**High risk**	**Low risk (%)**	**Medium risk (%)**	**High risk (%)**
PL1	−20,108 (−111)	−16,612 (−88)	−7,112 (−52)	−10	−13	−25
PL2^*^	−43,741 (−242)	−35,631 (−188)	−15,204 (−112)	−22	−28	−50
PL3	−58,623 (−326)	−47,223 (−250)	−20,298 (−149)	−29	−37	−60
PL4	−73,481 (−409)	−57,991 (−308)	−24,750 (−181)	−37	−46	−66
PL5	−87,804 (−489)	−68,904 (−367)	−29,628 (−216)	−44	−54	−72

* *PL2 is considered the existing level of prevalence in the UK (29)*.

Table 3 presents the percentage of farms that remain profitable (i.e., Z > 0) under alternative Johne's disease and farm support payment scenarios. In the table, columns correspond to increase in disease prevalence from left to right and rows represent with and without farm support payment scenarios. Under the “no-disease” scenario, 90% of the farms stay profitable. This means that 10% of farms are loss making even when there is no disease on a farm and they receive farm support payments. In the case of no farm support payment, proportion of profitable farms decreases to 80%. When the level of disease increases from PL1 to PL5; the percentage of profitable farms with farm support payment decreases from 90 to 76%. This reduction in percentage of profitable farms is relatively higher in the case when farm support payments are not available (i.e., 77–61%). This highlights the role of farm support payment under disease condition for farms to stay profitable. For instance, there are 86% (i.e., a reduction of 4%) profitable farms under disease level PL2 when farms continue to receive farm support payments compared with the “no-disease” scenario. In this case, all the farms that move from profitable to loss making under PL2 belong to the “high risk” category which is 15% of total farms in that specific financial vulnerability category. However, the percentage of profitable farms decreases to 72% (a reduction of 18%) when farm support payments are not available. This indicates that those 18% of farms that could stay profitable under PL2 disease level with farm support payments will become loss making farms when farm support payments are removed. The percentage of loss making farms in the “high-risk” category increased from 15 to 61% when farm support payments are removed under PL2 scenario.

**Table 3:** Percentage of farms (*n* = 50) each year with net profit greater than zero under various Johne's disease and farm support payment scenarios.

		**“No-disease” scenario (%)**	**Level of Johne's disease in a herd**				
			**PL1 (%)**	**PL2 (%)**	**PL3 (%)**	**PL4 (%)**	**PL5 (%)**
Farm support payments available?	Yes	90	90	86	84	82	76
	No	80	77	72	66	64	61

**Figure 4:**
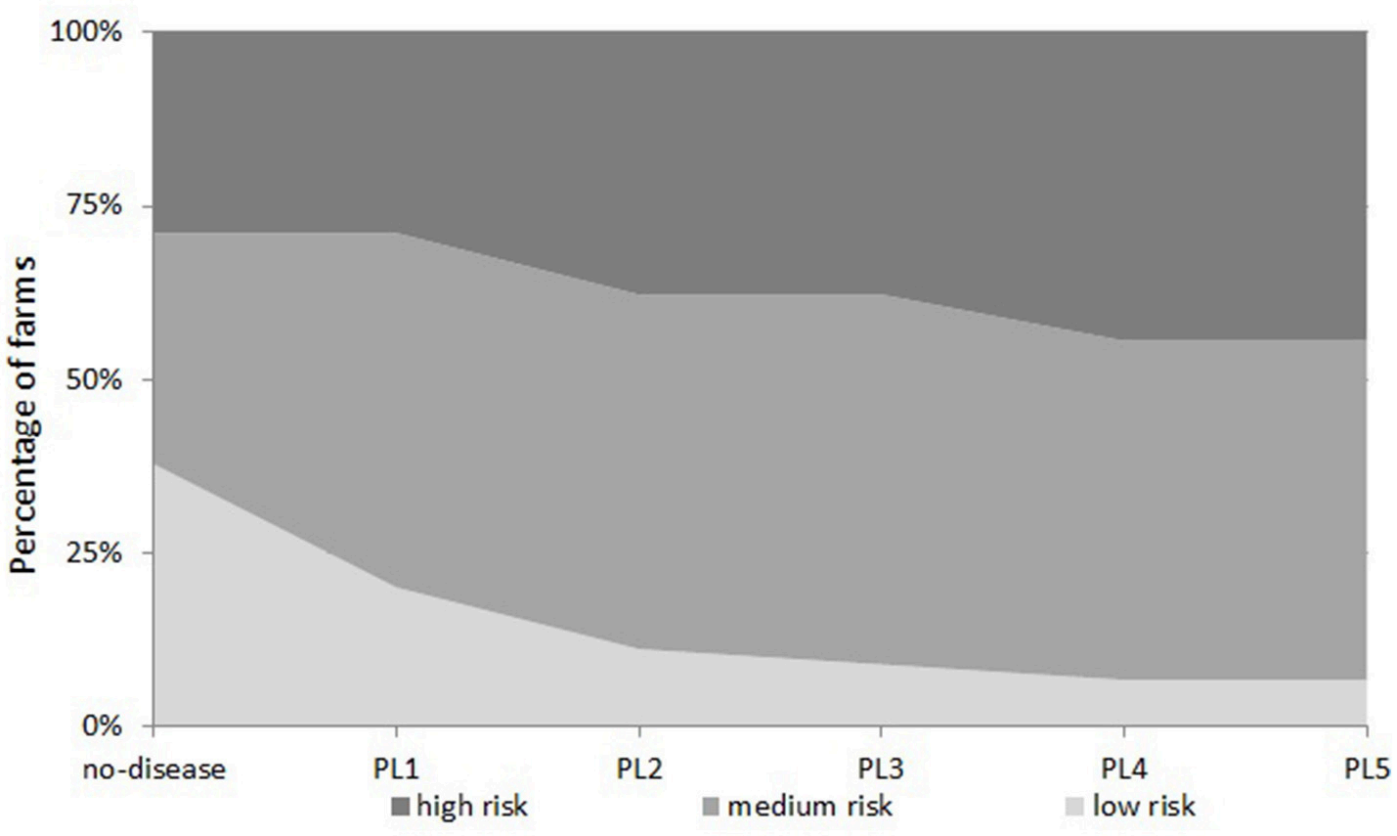
Percentage of farms in each financial risk category under five “disease” scenarios.

Examining the number of farms in all five disease scenarios together, we see that the proportion of farms in each financial vulnerability risk category is different in each disease scenario (Figure 4). This suggests that when disease prevalence level varies, the vulnerability risk status of a farm changes and farms move from one risk category to another. Under the “no-disease” scenario, there were 38, 33, and 29% of farms in “low risk,” “medium risk,” and “high risk” categories respectively. When the disease prevalence level is increased from PL1 to PL2, PL3, PL4, and PL5, a substantial percentage of “low risk” farms move to higher risk categories (45, 55, 65, and 65%, respectively). As Figure 4 shows, under PL5, there are only 7% of farms left in the “low risk” category but farms in the “medium risk” and “high risk” categories increase to 49 and 44%, respectively. On the other hand, reducing disease prevalence from PL2 to PL1 would increase the percentage of “low risk” farms by 9%; farms in “medium risk” stay the same but the percentage of farms in “high risk” categories would decrease by 9%.

Table 4 presents the averages of a selection of farm variables for profitable farms in the “no-disease” scenario as well as for the average farm in each of the three financial vulnerability risk categories. Among the farm variables, mean rough grazing area and farm support payment per cow were significantly different at the *p* < 0.01 level and milk yield per cow was significant at the *p* < 0.05 level between the three financial vulnerability risk groups. This suggests that major characteristics of farms in these groups can be differentiated by land capability, financial support and production level on farms. Specifically, farms in “low risk” group can be assumed to be high production level farms whereas farms in “high risk” category can be assumed as low producing extensive farms. The modeled “no-disease” farm net profit was different and highly significant between farms (*p* < 0.001) in the three risk groups.

**Table 4:** A selection of farm variables for an average Johne's free farm in each of low-risk, medium-risk and high-risk farm groups (5 loss-making farms in the “no-disease” scenario were not included).

		**Financial risk category**		
Farm characteristics Farm numbers	All farms (45)	Low-risk 17	Medium-risk 15	High-risk 13
**INPUTS^#^**				
Number of dairy cows	177 (83)	186	193	146
Stocking rate (livestock unit/ha)	2.21 (0.78)	2.52	2.22	1.75
Arable land (ha)	16 (29)	16	19	11
Grass land (ha)	140(69)	122	140	163
Rough grazing land (ha)^*^	21 (62)	4	16	47
Family labor (man labor unit)^*^	1.99 (0.72)	2.24	1.74	1.96
**OUTPUT^#^**				
Milk yield (liter/cow)^**^	7,182 (1712)	7,873	7,350	6,083
**COSTS^#^**				
Variable cost (£/cow)	237 (76)	257	250	196
Overhead costs (£/cow)	736 (187)	714	681	827
**PRICE^#^**				
Milk price received (£/liter)	0.22 (0.04)	0.23	0.22	0.22
**FINANCIAL SUPPORT^#^**				
Farm support payment (£/farm)^**^ Farm support payment(£/cow)^**^	35,011 (21,632) 219 (147)	27,910 152	35,640 187	43,573 342
**NET PROFIT^†^**				
“No-disease” farm net profit (£ ‘000/yr)^***^	141 (109)	216	138	48

*Figures between parentheses represent standard deviation*.

# *Farm Business Survey, 2016 data*.

† *Model result*.

* *significant at 0.05*,

** *significant at 0.01*,

*** *significant at 0.001*.

## Discussion

The model estimates of economic losses from Johne's disease presented in this paper are consistent with earlier studies. For instance, by extrapolating the results of this study, we estimate £185 loss per cow^3^ on an infected farm which comes within plausible range of £112 in the UK (4) and £46 to £192 in the US (1) per cow on an infected farm as estimated in earlier studies. Similarly, a Dutch study of Johne's disease estimated an average loss of £34,679 per infected farm per year (33) which is similar to our estimate of £31,940 per infected farm per year. However, lack of good estimates of prevalence levels is the main challenge to determining the economic impacts of Johne's disease. Many studies use different methods to determine prevalence levels. In this paper, we assumed a set of five prevalence levels and assumed prevalence of 17.5% to be the standard level. However, other studies have used different assumption; for example, the US study (1) used culling rate as a proxy for prevalence level whereas the Dutch study (33) used dynamic prevalence levels starting at 20% prevalence on infected dairy farms. More recently, a national economic welfare model (7) used a set of three prevalence levels (7.5, 17.5, and 27.5%) taking the national dairy population as a single herd.

Our results project that, if the farm support payments remain, only 4% of profitable farms in the “no-disease” scenario will become loss making under a standard disease level for the UK (PL2 disease level). However, 14% more once-profitable farms will become loss making farms under the same disease level if farm support payments are removed. This suggests that for these 14% farms, farm support payments provide a financial buffer against the disease. Under PL5, the highest disease level studied, 29% of farms move from profit making to loss making if farm support payments are removed compared to only 14% farms moving to loss making when support payments are included. Looking at the farm characteristics based on their financial risk categories, farms in “low risk” category receive significantly lower payments per cow than farms in the other two categories. However, these farms have a higher level of per cow milk production and also make significantly higher profit. Due to their higher production level, the absolute loss under disease scenarios is higher in these farms. However, because these farms stay resilient and still make profit under no farm support payment scenarios, they can afford to invest in animal health if required (13) as a risk management strategy to minimize their losses (34). However, farms in our “high-risk” category have lower levels of per cow milk production and are more reliant on farm support payments. A majority of these farms (61%) within this category will be highly vulnerable to the reduction or removal in financial support to these farms as a result of policy change, for example due to Brexit. If the disease level increases to PL5 level, then almost 30% of farms in the “medium risk” category will also become vulnerable to farm support payment policy.

As mentioned earlier, there are large uncertainties over estimation of disease parameters under different prevalence levels. This paper did not conduct sensitivity tests to examine the parameters and relied on earlier studies' results to estimate disease parameters. No activity on preventing the disease such as vaccination is included in this study. This paper also did not consider other incomes sources such as diversification and off-farm incomes which can arguably have an impact of farm financial status (35–38). However, as the focus of the paper was on economic impact of Johne's disease, it assumes farms to be specialized in dairy farming with no other income generating activities included.

## Concluding Remarks

Johne's disease has significant financial consequences on individual dairy farms as well as the national dairy sector. A majority of dairy farms are resilient enough to cover losses due to disease in addition to their other (non-Johne's related) costs or losses. However, around 14% of farms rely on farm support payment to cover their losses. These farms have lower per cow milk production levels, which are inadequate to cover economic losses from Johne's disease without payment supports. These farms are most vulnerable to changes in farm support payments and require attention when agricultural policies are reformed in the future or when designing and implementing a national control and eradication programme.

## Author Contributions

All authors contributed to the design of the work. SS and BV ran the model, performed the analysis and drafted the manuscript. All the authors contributed to and agreed with the manuscript.

### Conflict of Interest Statement

The authors declare that the research was conducted in the absence of any commercial or financial relationships that could be construed as a potential conflict of interest.

## Footnotes

^1^Details of the model are available here https://www.sruc.ac.uk/download/downloads/id/3513/scotfarm_%E2%80%93_a_farm_level_optimising_model.pdf

^2^Farms receive Pillar 1 (Basic Payment Scheme) and Pillar 2 (Rural Development Payment) under current EU's Common Agricultural Policy. Pillar 1 consists of farm direct payments which are the focus of this paper.

^3^To estimate the economic losses at the national level, we assumed the total number of dairy cows to be a single national herd and determined the losses under the PL2 scenario. The average net profit for all the farms at PL2 was £31,940 (SD ± 17,380) per year. Considering the total number of dairy cows to be 175,734 in Scotland (32), the average loss per cow per year and for all producers at the national herd was estimated at £185 (SD ± 100) and £5 M (SD ± 2.7), respectively.
